# Teachers’ negotiation of the cross-curricular concept of student digital competence

**DOI:** 10.1007/s10639-023-11800-x

**Published:** 2023-04-20

**Authors:** Christina Löfving

**Affiliations:** Department of Applied IT, University of Gothenburg, Box 100, Gothenburg, 405 30 Sweden

**Keywords:** Digital competence, Digital citizenship, Teaching, Sensemaking, Boundary object

## Abstract

In a digital society, teachers are required to carry out policy directives on both core knowledge and more vaguely described cross-curricular competences, one being digital competence. This paper reports on the findings of a study in which 41 teachers from three lower secondary schools in Sweden engaged in focus group interviews where they participated in sensemaking processes on students’ digital competence. The questions targeted what the teachers knew of their students’ digital experiences and how to facilitate and further develop these students’ digital competence. Based on the focus group interviews, four themes were identified: *critical awareness*, *tool management*, *creativity*, and *avoidance* of digital usage. Absent were themes related to democratic digital citizenship. The paper discusses the importance of moving away from a one-sided focus on individual teachers’ professional digital competence in favour of focusing on how school organizations can negotiate and facilitate students’ digital competence in local situ. Otherwise, there is a risk of overlooking students’ cross-curricular digital competence and digital citizenship. This paper is a starting point for further research on how school as an organization can support teachers in facilitating various areas of students’ digital competence in a digital society.

## Introduction

Digitalization has been part of society, and school, for a reasonably long period of time. At the same time, the narrative of school digitalization provided by, for example, policymakers and Ed-tech initiatives has been enthusiastic about what can be achieved in education and what competences are needed. Even if there are narratives of both success and the lack thereof, what tends to be left out is the complexity of education (Pettersson, 2018; Sancho-Gil et al., 2020; Selwyn, 2011), a complexity magnified by the COVID-19 pandemic, which affected teachers’ roles and professional digital competence as education was primarily conducted online (Li & Yu, 2022). The crisis underscored the feasibility of online teaching as opposed to focusing on the development of new pedagogical ideas or reflection (Ewing & Cooper, 2021). Some years have passed since the outbreak of the COVID-19 pandemic, and therefore, the time has come for further research on what can be the new normal in a society where digitalization has become part of life and where teachers and students try to find their way.

Education concerns not only core knowledge and skills; it also initiates students into various contexts and impacts how they come to exist as persons (Biesta, 2015). In the digital development of recent years, the concept of *digital competence* has been emphasized in educational policy documents in several countries (Ala-Mutka, 2011). In a digitalized society, certain competences are required and (in some countries) concern democratic skills and values framed as digital citizenship (Choi, 2016). However, while students grow up getting used to using digital technology, their experiences might conflict with how teachers perceive the way in which such technology is supposed to be used in teaching practices or with what they regard as essential teaching (Aagaard, 2015). These studies suggest a conflict between students’ experiences and how teachers choose to facilitate the development of digital competence, which can add to the complexity of teachers’ work. Chou and Block (2019) previously reported on the gap between teachers’ intentions and students’ expectations, where teachers tend to allow students to use digital technology for reading or writing and focus on information seeking as part of digital competence at the expense of collaboration, problem-solving and creativity. Building on the work of these earlier studies, this paper examines how teachers in lower secondary schools in Sweden engage in sensemaking processes on students’ digital competence. This process is particularly relevant in the context of Sweden where digital competence is explicitly mentioned as a cross-curricular concept in the national curriculum (National Agency for Education, 2022b) and where the Education Act stipulates that teachers participate in pedagogical, collaborative work to ensure that objectives in education policy directives are achieved (SFS 2022:1319, Chap. 4, 4–5 §). Given these conditions, this paper considers teachers’ understandings of students’ digital experiences as an important part of teachers’ sensemaking processes. These experiences are here framed as students’ net-based out-of-school activities, acknowledging that all online activities are also conducted as part of physical life-worlds or, as Floridi (2014) put it, onlife. Against this backdrop, this paper asks as follows: How do teachers engage in sensemaking on the cross-curricular concept of digital competence in relation to their students’ net-based out-of-school activities?

## The concept of digital competence as part of teaching practices

There have been various trends in digitalization research regarding what to focus on and what concepts to use. In the early days, the computer itself was in focus. This was complemented by analyses of computer literacy as an important concept, followed by those relating to information. During the first decades of the 2000s, digital literacy concepts such as 21st century skills (Ananiadou & Claro, 2009) and digital competence came into play in research, especially in relation to policy (Stopar & Bartol, 2018). One of the first to map the latter concept was Ala-Mutka (2011), who stated that it concerned aspects of knowledge, skills and attitudes. This paper does not aim to provide a thorough description of exactly how the concepts are used (see Martínez-Bravo et al., 2022) but will highlight some commonalities and variations, especially in the Nordic countries since the study setting is in Sweden.

Policies are supposed to regulate education in specific directions (Ferrari et al., 2012). Consequently, policies on digital competence from the Organization for Economic Co-operation and Development (OECD, 2021) and the European Commission (EC) (European Commission et al., 2022) can be understood as seeking to have an impact on educational systems. In *Europe’s Digital Decade: Digital Targets for 2030* (European Commission, 2021), digital citizenship and democratic values are emphasized by, among other things, promoting individuals to participate in digital democratic spaces where they can be empowered. When digital competence is defined as distinct from information and data literacy, as in the European Digital Competence Framework for Citizens (DigComp 2.2), there are also aspects of participation in digital spaces connected to collaboration, creation, safety and problem-solving (European Commission et al., 2022). However, policies are seldom specific or concerned with details, which is why they need to be interpreted contextually (Ball et al., 2012). Olofsson et al. (2021b) investigated the varying interpretations of digital competence at the national level in Norway, Sweden, Finland and Denmark due to the plasticity of the concept. In these Nordic countries, policies are interpreted differently at the local level due to the context. Taking Sweden as an example, factors such as resources and the size of school organizations matter, thereby justifying further research on the views of organizational participants (Gustafsson, 2022), an important contribution of this paper.

Norway became the first Nordic country to emphasize digital competence as a key competence, with *bildung* being an essential part, in the national curriculum of 2006 (Krumsvik, 2008). Krumsvik stressed that “Digital bildung [digital danning in Norwegian] focuses on how pupils’ participation, multi-membership of different communities and identity development in the digital era are influenced by the digitization of society” (2008, p. 288). This relates directly to the democratic values identified in European policies (European Commission, 2021; European Commission et al., 2022). Additionally, students are to use digital technology to reach competency goals both across and within various subjects (Olofsson et al., 2021b). In Sweden, democratic values are emphasized in the national curriculum (National Agency for Education, 2022b), and the concept of bildung is also expressed there, albeit not clearly in relation to digital competence. In the Swedish national curriculum, digital competence is generic and cross-curricular, similar to the national policy of the Swedish government: “digital competence implies that everybody should be familiar with digital tools and services, and have the ability to participate in digital development based on their prerequisites [here translated from Swedish]” (SKR 2017/18:47, p. 8). In 2022, a new national strategy on digitalization in education (National Agency for Education, 2022a) was proposed in Sweden. One of its objectives was that all students were to “develop digital competence to be able to actively participate in studies, society, and future work life to contribute to a sustainable and democratic society [here translated from Swedish]” (p. 7). However, the main focus in the Swedish syllabi of subjects concerning digital competence is on operational and tool-oriented understandings while critical aspects are mainly represented in school subjects concerned with the evaluation of information and sources (Godhe et al., 2020). Digital competence is sometimes used in combination with the adjective *adequate* in Swedish policies, emphasizing the need to negotiate what is adequate at different times and regarding the pace of change related to it (Erstad et al., 2021). Thus, *adequate* signals that the concept cannot be defined once and for all and needs to be continuously interpreted due to changing circumstances and societal contexts (Fransson et al., 2018). In Finland, the concept of information and communication technology (ICT) is used instead of digital competence and mainly concerns technical usage, information searches for inquiries and creativity and interaction with others (Olofsson et al., 2021b). In Denmark, digital competence is framed as the use of digital technology to enhance STEM (science, technology, engineering and mathematics) skills and digital citizenship in a democratic society (Olofsson et al., 2021b). Norway, Sweden and Finland have decided to incorporate programming into some subjects of their national curricula (Erstad et al., 2021), though with some variations. In Finland, programming is part of mathematics and crafts, while in Sweden, it is part of mathematics and technology (Heintz et al., 2017; Mannila, 2018). In Norway, programming has recently become part of mathematics, natural science, music and arts and crafts (Kravik et al., 2022).

Nevertheless, while digital competence is continuously framed in various ways in the above-mentioned Nordic countries, they all embrace it as connected to both core knowledge and how to be a citizen in a democratic society. However, according to Lund (2021), there is a risk that democratic values and activities in a digitalized context are downplayed in policies, while standardization and tool-oriented and instrumental skills are emphasized.

## Facilitating student digital competence

Teachers in the Nordic countries are allowed a high degree of self-determination to interpret policies in their local teaching practices (Klette, 2002); the interpretation of policies on digital competence and what is adequate to teach can be understood as part of Nordic teachers’ self-determination. The European Commission put forward a Digital Competence Framework for Educators, which targets professional digital competence (PDC), that is, what it means for teachers to be digitally competent (Redecker & Punie, 2017). The competences in the framework include professional engagement, pedagogic competences concerning teaching and learning, digital resources, assessment, empowering learners and teachers’ ability to facilitate learners’ digital competence.

Lately, research has focused on teachers’ PDC, strategies and struggles, especially concerning teachers in upper secondary schools and higher education (Howard et al., 2021; Ogodo et al., 2021; Olofsson et al., 2020, 2021a; Scherer et al., 2021). Less common is research on how teachers in K–12 schools, as part of teachers’ PDC, try to understand their students’ digital competences in order to facilitate and develop them further (Richardson et al., 2021). However, when teachers have opportunities to negotiate how to facilitate students’ digital competence, the discussion tends to be tool-oriented and does not focus as much on democratic aspects or the skills needed in a digital society (O’Neal et al., 2017). Teachers’ emphasis on facilitating students’ digital information and communication skills – which can be considered part of teachers’ PDC and as aiming to develop students’ digital competence – is positively related to teachers’ ICT skills, self-efficacy, frequency of ICT use and perceived usefulness of ICT (Siddiq et al., 2016b).

However, there are challenges related to teachers’ PDC. When social structures transform due to digitalization, they might feel less confident about their work (Collin et al., 2018). In a highly digitalized society, students are often able to conduct immediate accuracy checks on the Internet regarding the knowledge provided by teachers and are often used to more spectacular online content, something that teachers find difficult to provide (Löfving et al., 2023). Teachers also report of contextual barriers such as lack of equipment, support and time, making the contextual environment important (O’Neal et al., 2017). If the above-identified barriers have an impact, questions can be raised regarding what teachers would discuss if some of the barriers were to disappear. Importantly, in Sweden, where students from lower secondary school and onwards are often equipped with one digital device each by their school, lack of equipment is not a major concern.

Another challenge for teachers is to know what to focus on and how to facilitate digital competence. When students are asked about their digital experiences, the questions tend to focus on some aspects while neglecting others (Siddiq et al., 2016a). Digital competence is often discussed in connection with whether students are passive and vulnerable adolescents and the need for reductive initiatives for cyber safety. These initiatives seldom respond to the variety of student experiences or attend to questions about their rights to safety and belonging (Black et al., 2022; Collin et al., 2018). One aspect discussed is the amount of time students spend online. In a large-scale survey, more than 500 secondary school students were asked how much time they spent on non-academic ICT (NA-ICT). The amount of time was compared with their school achievements, showing a negative correlation (Salomon & Ben-David Kolikant, 2016). The researchers’ suggestion was that students needed to learn when to focus on learning and when to relax using NA-ICT instead of only being told about what could be dangerous on the Internet, which, the researchers stated, was common in schools. In another large-scale study with more than 4,000 upper secondary school students, their out-of-school experiences concerning academic and non-academic usage and digital competence were found to be diverse. Therefore, it is crucial for teachers to pay attention to this diversity when trying to facilitate digital competence in their teaching practices (Hatlevik & Christophersen, 2013). Livingstone and Third (2017) argued that there is a tension between “children’s right to participate and to be protected online” (p. 662) and that various stakeholders promote either participation or protection. They referenced the United Nations Convention on the Rights of the Child and the European General Data Protection Regulation in their criticism of both society and researchers for not acknowledging children’s rights. These rights should, according to them, concern privacy, safety and development, not just on a universal level but as part of children’s everyday lives. Even so, when digital technology enters the teaching practice, its benefits mainly relate to the logistics of school work rather than aiming to enhance learning or developing various skills (Selwyn et al., 2020). Based on these studies, one might argue that even if students have easy access to digital technology in school and their net-based out-of-school activities, as well as some operational understandings, this may not fulfil the expectations for sophisticated digital competence in the sense often implied by the concept of digital citizenship.

## Methodology

### Research context

Sweden is an ideal example of a research context where the concept of digital competence has been evident in the national curricula of recent years, namely, since 2018 (National Agency for Education, 2018). During the autumn of 2021, 41 teachers representing all Swedish compulsory school subjects were interviewed in focus groups. The reason for choosing this method was to gain knowledge on teachers’ collective sensemaking at schools where teachers share responsibilities for students and facilitate their cross-curricular competences. The interviews were conducted in five groups in three Swedish lower secondary schools on the west coast of Sweden and lasted 55–75 min (total = 340 min). Schools A and B are situated in small villages in rural surroundings, where the socioeconomic status is considered slightly below average, while School C is situated on the outskirts of a large city, where the socioeconomic status is considered high. The teachers all knew each other and were used to work-related discussions. They were mainly experienced teachers who had used digital technology in education for several years, and their students had easy access to digital devices. Before the interview, they had undergone pandemic-related online teaching where the focus was mainly on getting digital platform teaching to work. The teachers will here be referred to by their school’s name (A, B or C), and a number A = *n15*, divided into two groups (*1–8*, *9–15*), B = *n22*, divided into two groups (*1–11*, *12–22*), or C = *n4*, one group (1–4). The reason for the pseudonyms is the ethical refrain from revealing identities. Because of the cross-curricular focus, their specific school subjects were not of particular interest.

### Preparations and focus group interviews

The teachers were informed in advance in a letter of consent that the focus group interview would be a discussion, rather than an interview with only pre-decided questions, on students’ net-based out-of-school activities and that the discussion would target the relation to the teachers’ teaching practices since digital competence is part of the national curriculum. At schools A and B, the principal decided that the teachers would participate as part of their professional learning; at School C, only four teachers decided to participate.

At the beginning of the focus group interviews in schools A and B, the teachers had a few minutes to write down reflections on the question: *What net-based activities do you think your students practice out of school?* One group (School C, where only four teachers participated) decided not to write anything and to start the discussion right away. The teachers then shared similarities and contradictory understandings. The next question was *In what ways, if any, do you think these activities affect teaching?* There were follow-up questions on *how the teachers try to facilitate what they had identified as necessary for their students to develop further*. Asking questions about students’ net-based out-of-school activities was supposed to facilitate broad negotiations on digital competence connected to the students’ experiences. The schools and the interview situation have previously been described in another paper with another focus (Löfving et al., 2023).

### Ethical considerations

Some weeks in advance of the focus group interviews, the teachers were informed through their principals that I, the researcher, had asked for access to their schools. The principals then forwarded a letter of consent to the teachers, which followed the guidelines of the Swedish Research Council (2017). Even if the principals of schools A and B had decided that the focus group interviews would be part of the teachers’ professional learning, I made sure that it was voluntary to participate, both in the letter of consent and when I met the groups. The principals were not supposed to attend the interviews and did not do so. Before signing the letter of consent, I asked the teachers whether they had any questions and whether they wanted to participate. Even if I made sure that no names were mentioned in the paper deriving from the study and that participation was voluntary, I could not be certain whether anyone felt obliged to participate, which is why I stressed the opportunity to contact me afterwards if they wanted to withdraw. No one did. Another consideration was how I steered the questions to start with the students’ net-based out-of-school activities, something that the teachers might not have felt was part of their work. However, I tried to let the teachers choose topics of interest to them in relation to the questions in order to facilitate their negotiations rather than steering them away from what they found to be relevant to negotiate as part of their work.

### The analytical concept of sensemaking

According to Weick (2001), organizations are not just buildings or policies; they also constitute places where actions and negotiations occur as part of sensemaking. Using the analytical concept of sensemaking, Weick et al. (2005) elaborated on how people try to make sense of the world while drawing on their understandings – their so-called maps. When people come together and share their maps, new understandings might evolve. In an organization such as a school, where teachers share their experiences, such sensemaking is part of work – sharing and negotiations lead to a sort of justification of one’s values, which are deeply connected to how one acts (Weick et al., 2005). Thus, participating in the process of sensemaking on policies could be understood as a way for individuals to display their agency (Ball et al., 2012). When faced with complex situations, collegial negotiations could offer teachers opportunities to better understand what sometimes begins in solitude and action (Johnston & Fells, 2017; Weick et al., 2005). The concept of sensemaking will therefore serve as an analytical tool when inductively studying how teachers negotiate their understandings of the cross-curricular concept of digital competence. Furthermore, the focus group interviews will be referred to as discussions since they contained both statements and negotiations.

When a vaguely described concept, such as that of digital competence, is targeted in the sensemaking process, it might evolve into a boundary object (Star & Griesemer, 1989). An *object* can be understood as something that people act with and towards. It is not just a dead thing. A boundary object can be understood as “plastic enough to adapt to local needs and the constraints of the several parties employing them, yet robust enough to maintain a common identity across sites” (Star & Griesemer, 1989, p. 393). Since not all teachers have the same assignments, they seldom relate to a shared object in the same way, hence the notion of plasticity. When their understandings of the object meet, conflicting ideas can appear. Consequently, when an organization needs to create shared understandings, there are often tensions between divergent viewpoints rather than a shared consensus (Star & Griesemer, 1989). Other essential recognition markers of boundary objects are that they are temporal, local and subject to reflection. Further, they consist of several dimensions (Star, 2010). Star and Griesemer (1989) stressed the need for people to communicate and negotiate in order to allow such boundary objects to emerge. This contradicts the view of work as simply acting upon instructions in a predefined manner. Instead, boundary objects serve as temporary bridges that facilitate autonomy, heterogeneity and cooperation. However, cooperation can also facilitate less preferable situations, such as silencing and fragmentation (Star & Griesemer, 1989). When standardizations emerge, new questions will appear, developing new boundary objects (Star, 2010). Even if Star and Griesemer (1989) did not mention the concept of sensemaking, what they described share commonalities with the concept and help understand how it concerns people perceiving objects somewhat differently and the meanings on which they can agree, at least in part. Olofsson et al. (2021b) previously used the concept of boundary object to analyse “how digital competence can provide the possibility to trace signs of how intentions at the transnational policy level are translated, negotiated and inscribed into Nordic national policy” (p. 318). In envisioning the concept of digital competence as a boundary object where teachers can share some understandings and where other understandings can be reconsidered and perhaps transformed, the concept can be understood through the analytical lens of sensemaking. With such a lens, teachers’ discussions regarding areas where their various personal understandings are illuminated and negotiated are of particular interest.

To inductively analyse the aspects of the boundary object of digital competence regarding which teachers share some understandings, thematic analysis was conducted in accordance with the methodology of Braun and Clarke (2006). Following their six-step methodology, the five recorded interviews were (1) first transcribed by me [the author]. Thus, I was able to familiarize myself with the data. The transcriptions were then uploaded to the NVivo programme, and the entire data set was read several times as some initial general ideas emerged, for example, that the teachers, among other things, seemed to discuss their students’ physical well-being, which were noted down. (2) To generate initial codes, the transcripts were marked with different colours on a manifest level across the entire data set due to various identified aspects, such as what net-based out-of-school activities the teachers thought their students were involved in and how they said they could facilitate their students’ needs. (3) Subsequently, the various codes found throughout the entire data set were compared, looking for commonalities, or themes, on a latent level. One prominent theme emerging was digital content, that is, teachers’ concerns about the digital content that students have access to during their net-based out-of-school activities and how they manage it. (4) This particular theme was then reviewed and repeatedly compared with the codes of the entire data set. (5) The theme was initially named *developing technical skills*, later renamed *digital content*, while other themes were named *creativity and avoidance.* Initially, the theme renamed *digital content* consisted of four sub-themes. Following later comparisons and discussions with others, it was divided into two themes, *critical awareness* and *tool management*, with no subthemes, to enhance clarity. (6) Finally, excerpts defining the themes were chosen to provide an understanding of the themes that would help answer the research question. Since a larger number of teachers from School B (*n22*) participated, a lesser number from School A (*n15*) and an even lesser number from School C (*n4*), the number of excerpts from each school reflects this distribution.

## Results

The study sought to answer the following research question: How do teachers engage in sensemaking on the cross-curricular concept of digital competence in relation to their students’ net-based out-of-school activities? Teachers discussing their students’ net-based out-of-school activities helps draw a picture of how they perceive the boundary object of digital competence while taking their students’ experiences into consideration. The themes identified in the data were *critical awareness*, *tool management*, *creativity* and *avoidance*.

### Critical awareness

The teachers had noticed how the students used social media as part of their net-based out-of-school activities to keep up with contemporary culture through influencers and post pictures and video clips. Additionally, the students were thought to use social media to find tutorials, learn things and get information about what was happening in the world. They seemed to practice English when communicating extensively with others and to use online dictionaries to solve language difficulties. Much time was spent on the Internet searching for items to buy or playing games. There were notions that it was problematic that the students were retrieving information from social media: “They do not see the source!” (B19). The teachers discussed that the information that the students encountered was often pre-processed by others: “They get it [the information] from biased sources.” (A11). However, the teachers were not sure which sources the students had encountered but saw critical awareness as essential in both school and out of school. Even if the teachers stated overall that the students encountered questions relating to critical awareness in most school subjects and, therefore, knew how to be critical towards historical sources, they needed to further develop critical awareness in their everyday lives: “They aren’t as critically aware when they communicate with each other or get to know about a rumour or something that has happened in the neighbourhood.” (C2). Another teacher from another school reflected: “I reckon they are really good at critical awareness when it comes to schoolwork, but it becomes something else when they see something not connected to reality, which [reality] is what we focus on in school.” (B6). These concerns were raised across the board, regardless of the teachers’ subject area.

#### Facilitating critical awareness

The teachers agreed that critical awareness was brought up in almost all school subjects in one way or another. However, none of the schools discussed commonalities between the various subjects. During the discussion, they agreed that they rarely taught how to be critically aware of information gained through social media, except in relation to historical documents. In School B, they provided some examples: “First-hand source, second-hand source, etc.” (B11). When asked how the teachers considered the information sourced by students from social media, the answer was, “We question them, of course. You have to prove that it is a good source.” (B15). One of the teachers (B12) provided an example of when some students asked whether it was true that some of the core contents in the physical education syllabus were going to be removed since the students had heard this from a teacher on TikTok (a social media platform). The teacher (B12) then asked the students whether the teacher providing the information was “a good teacher”. When the students did not know, the discussion was closed. “But it was so obvious that they had not checked the source. They had just reacted on the feeling that they thought it was great that it was going to change.” (B12). Questioning the source and closing the discussion here served as an example of how the teacher approached the aspect of critical awareness.

### Tool management

According to the teachers, the students seemed to use digital devices frequently in their net-based out-of-school activities. They also brought their net-based out-of-school activities into the classroom, sometimes straying away to play games or to try to purchase items online: “I think that there is quite a lot of online shopping going on.” (B14). Even if the teachers were concerned about the content that students might come across when using digital devices (here labelled tool management), one of them stated that when students use websites and applications “[y]ou get access to knowledge very quickly, knowledge you wouldn’t search for in a textbook or so.” (B18). One teacher from School C had a similar reflection: “But the next step is to know how to use the knowledge. And maybe that will be another way of learning.” (C2). Even if the students seemed to know how to manage their digital devices for net-based out-of-school activities, according to the teachers, they lacked some competence in structuring digital content, for example, “structuring files and knowing where things are.” (B15) and managing it for schoolwork: “Some of them cannot open their emails and don’t know how to get access to them.” (A2). Furthermore, the teachers from School A discussed whether the students had gained deeper knowledge on how digital technology work underneath the surface, for example, how the templates that help students write text documents, tables and presentations actually work: “I think that it is essential to understand the foundation of it. […] Otherwise, you lose something on the way. Or that is what I imagine.” (A2). Another teacher (A7) raised the question of whether enough attention was being paid to teaching the students these things.

The teachers stressed that students also needed to develop competences regarding search techniques, not necessarily needed out of school but for schoolwork: “I also have the experience that they are quite lousy at searching for further information. If they do not get results immediately, they give up.” (A13). The teacher described how the students immediately asked teachers for help when they could not find information in the digital textbooks or teacher-provided material: “There are too many clicks, and ‘where can I find this?” (C4). The examples were both related to retrieving information from the Internet and finding information in digital textbooks or teacher-provided material. Nevertheless, the students seemed to prefer to find the information needed for schoolwork from sources other than the textbook provided: “If they get an assignment to answer some questions, then you can almost be sure that the answer comes from Google instead of the textbook.” (A2). Thus, the teachers raised the issue that access to search engines is a challenge for teaching, although they did not pursue the discussion further.

#### Facilitating tool management

When asked how the teachers tried to facilitate the students’ competences regarding managing and structuring files in folders on the computer, something the teachers identified as essential, the answer was, “Well, that is a good question!” (B15). A teacher from School A expressed that it was continuous work: “You have to show them.” (A2). Another teacher filled in, “Again and again.” (A8). When asked about the identified need for students to learn what is behind digital templates and how the teachers would approach this in their teaching, there was no answer and, thus, no elaboration.

As the teachers acknowledged that students could have a difficult time finding relevant information, they wanted to broaden their students’ knowledge by providing them with such information: “Introducing them to good websites can result in them using them later; I can imagine that some [students] have done that.” (A13). However, a teacher from School A (A9) preferred to be the thesaurus instead of letting the students find translations on the Internet since the other students then also got to learn the answer. A teacher from School C (C3) solved the problem of searching for information in digital textbooks by providing the students with direct hyperlinks to necessary information. In some subjects, the teachers used textbooks instead of digital books to solve the problem of navigating on the computer. However, how to search for relevant information using digital devices was done differently in different subjects, depending on what the teachers found helpful: “No, we haven’t discussed that with each other, no.” (A13).

### Creativity

The teachers provided a variety of examples of students’ creative competences deriving from sources that appeared diffuse to the teachers but which they reckoned were connected to the students’ net-based out-of-school activities – for example, students deciding to learn how to play the piano or knit by following a tutorial on the Internet or trying their best to make artistic notes: “[and] they might have got the inspiration from TikTok or somewhere else.” (C3). However, other students did not seem to have the same inspiration: “[when] some write with a pencil… You cannot read what it says.” (A8). There were examples of the students’ out-of-school creativity while editing photos and posting video clips when dancing as well as notions that this would change since, according to the teachers, popular culture was constantly changing. Some students were deeply engaged in their activities, which a teacher (B15) spoke of as the spirit of Greta Thunberg, a young Swedish environmental activist. Even so, the teachers were worried that the students were not highly creative: “But it is rather one-sided for the one who is stuck in it.” (B13). Some emphasized that students mostly seemed to be consumers watching film clips and playing games, not producers, which the teachers reckoned was problematic. During breaks, “[up] to 40 students can be sitting there, all of them are looking down at their phones.” (C4). Such seemingly passive usage was deemed a hinderance to creativity.

#### Facilitating creativity

There were notions that the teachers were limited in how to influence the students’ creativity: “I don’t think they will go home and think, ‘now I would like to do a PowerPoint presentation about this’ (laughs).” (A8). Instead, all three groups of teachers elaborated on how to draw on the students’ experiences and turn them into writing assignments, engage them in their learning and be creative doing schoolwork. Using digital applications with a certain amount of competition was thought to attract students and engage them. A teacher from School C (C4) described how a digital game could motivate students to learn the names of some countries more than learning the same from a book. However, another teacher (C3) at the same school reflected on how competition could increase stress among students. In School A, a teacher had another approach and published tutorials made by the students on a website. Therefore, “the students get to practice how to share information with other students. […] We combine their experience with mine, in a sense.” (A12). Nevertheless, publishing on the Internet as part of teaching was not evident in the other teachers’ answers.

### Avoidance

The teachers were concerned about how much time their students spent on net-based out-of-school activities, with the digital aspects seemingly consuming too much of their time: “They don’t feel as a whole person without their phone” (A14), and “The notifications [on the phones] sound all the time.” (B20). There were concerns about algorithms steering the students in directions where they could not control themselves. Lately, the teachers had become aware that the students constantly knew where their friends were since they followed each other on the Internet. There was a coherence in the narratives that the students needed to learn how to avoid such activities. Furthermore, the students were thought to benefit from such avoidance: “I think that there is a need to be able to do things with the hands […].” (C2). This last statement is in line with thoughts expressed by other teachers, for example, a teacher (B7) from School B stressed that students needed to avoid copying and pasting text from the Internet and, instead, write using pencils.

Furthermore, there were concerns about students bullying each other online and how to prevent such behaviour. The harassment was more of a feeling since the teachers discussed that they did not know everything that their students were involved in, even if the students sometimes told the teachers about it: “There are so many things we never get to know of and which the kids keep to themselves because they have so many hidden groups [on the Internet] that we don’t…” (B10). There were also concerns about students watching pornography online, which the teachers overheard from the students themselves: “You hear the jargon between them, so to say.” (A11). All these examples target discussions of avoidance. The concerns were based on both what the teachers noticed about the students’ net-based out-of-school activities in the teaching practice and what they believed was happening in their students’ life-worlds.

#### Facilitating avoidance

Teachers from all three schools provided examples of wanting to help students avoid the digital sphere in some sense or focus on other aspects since “We also need to contribute to something other than what they encounter in their world; otherwise, we do not provide the knowledge assignment we have as teachers.” (C4). Some of the teachers expressed that they felt pressure to relate to students’ previous experiences in their teaching, and a teacher (B5) from School B stated that it might be good to separate school from students’ net-based out-of-school activities. A colleague concurred: “Can’t they just have their TikTok? Because, sometimes, it feels like ‘now you have to come up with a suggestion of how to use TikTok during lessons since they are using it.’” (B6). A teacher from School A supported this notion: “I try to use it [digital devices and applications] in a way that they will understand that we can use it for purposes other than TikTok.” (A12).

The specific problem of copying and pasting text was sometimes solved by letting the students use pencils and papers. As a teacher noted, “I want them to have their own [handwritten] notes, to own their material. To just copy and paste from the Internet, that is…” (B7). As the teacher further stated, “Since we know that there is a strong correlation between learning and the hand and brain.” At School C, information about how the brain works was thought to help students avoid too many net-based activities where the digital influence was strong.

Even so, the teachers were unsure as to how to facilitate some of the students’ need for avoidance of, as they explained, being tracked by algorithms. Furthermore, some of the issues identified as problematic digital usage included tracking friends through various applications and constantly knowing friends’ locations, something that some of the teachers did themselves: “That was why I checked my phone right now to see if my kid had arrived home […].” (B12). Practicing avoidance in these areas raised new questions: “I have learned that you can *do* things, maybe not on TikTok, but they don’t get the chance to break free. How do they do that?” (B12). The question was left unanswered. When the teachers from School B were asked whether they discussed these pedagogical issues at other times, one of them stated, “Maybe it hasn’t come up naturally.” (B5). Others continued, “Maybe we talk about what they are not supposed to do on the computers.” (B11); “Yes, limiting their use. That is what we work a lot with, I guess.” (B4).

Another issue that concerned the teachers in relation to avoidance was bullying others online. When such bullying occurred, it was mainly met by discussions between the teacher and students. At School C, according to a teacher (C1), these discussions were dedicated to specific lessons about health issues. There, the teacher and students, on some occasions every year, talked about healthy online behaviour. According to teachers from schools A and B, access to pornographic material was also discussed with the students during specific lessons in natural science.

## Discussion

The results of this study unpack how teachers from three lower secondary schools in Sweden negotiated digital competence as a cross-curricular boundary object as they discussed students’ digital experiences. They add to earlier work on how digital competence can be understood as a boundary object at the national level in Nordic countries (Olofsson et al., 2021b)by examining local school contexts. The analysis produced four themes that can be understood as overarching in the sense that they are cross-curricular and independent of the subjects that teachers teach: (1) critical awareness, (2) tool management, (3) creativity and (4) avoidance. Across these themes, the analysis raised several key issues that warrant discussion.

First, the results showed that all the teachers encountered questions on critical awareness, whether they taught physical education or other subjects, and needed to act on these issues. Even if they did not believe that the students were critically aware of what they learnt on social media, they lacked examples of how to cater for or further develop this competence. The teachers acknowledged, as Black et al. (2022) had before, that education in critical awareness tended to be one-sided when focusing on historical sources, but they then went on to discuss other matters. Additionally, they expressed uncertainty about handling this topic beyond questioning the sources connected to the students’ net-based out-of-school activities.

Second, structuring files and retrieving information are part of cross-curricular tool management, which is a necessary part of digital competence, according to the teachers. They stated that the students had not gained enough experience in these areas from their net-based out-of-school activities, with each teacher deciding how to further facilitate such competence within the context of schoolwork. This resulted in continuously reminding students of how to explore digital issues that each teacher found important for their school subjects. Thus, digital competence was discussed as cross-curricular but as something each teacher encountered differently. The students, therefore, had to adjust to the teachers’ various PDCs rather than develop their “ability to participate in digital development based on their prerequisites” (SKR 2017/18:47, p. 8). Additionally, while focusing on tool management, questions on emancipation and other aspects tended to be neglected, as previously reported on by Chou and Block (2019).

Third, there were concerns about the students’ creativity, that is, they were not sufficiently creative and consumed the materials of others rather than producing content themselves. These concerns could be regarded as connected to what Krumsvik (2008) referred to as digital bildung, which has to do with growing as a person when using digital means – something the teachers stressed that they had not noticed much of. Nevertheless, they provided examples of creativeness, for example, students learning how to knit and write artistic notes from their net-based out-of-school activities. What was evident was that the teachers did not think that the students would appreciate what they as teachers could provide, neither would they use the knowledge and skills learned in school in their out-of-school activities. Thus, the teachers questioned their own influence on empowering students in their everyday lives. This can be an answer as to why the teachers did not provide many examples of how to act on what they had identified as adequate digital competence among students in a digital society. When the teachers described the students’ experiences, it was mainly to gain motivation for the core content of the syllabi of subjects. Nevertheless, they negotiated creativity as part of the boundary object of digital competence, even if they were unsure of how to facilitate it further.

Fourth, the teachers were preoccupied with how to facilitate avoidance of the digital realm when needed, making avoidance an essential part of the boundary object of digital competence. There were concerns about the unsuitability of specific social media applications for education. The students’ use of their lesson time to do online shopping was seen as a hindrance to essential knowledge, not something that students needed to handle critically. They tried to provide content other than what the students encountered in their net-based out-of-school activities so as to nurture their knowledge development. However, absent were discussions on how to teach democratic values in a digital society and being an active youth navigating a society full of digital applications without avoiding them.

What is striking is that the teachers reported that they seldom negotiated students’ experiences related to digital competence, neither as a cross-curricular issue nor as a subject-specific one, and how to further facilitate their competence. However, during the discussions, they negotiated various viewpoints which created a nuanced picture of how they perceived the boundary object of students’ digital competence in relation to their net-based out-of-school activities. Overall, the teachers participating in the sensemaking process were experienced, and their main challenges were not connected to lack of equipment, as O’Neal et al. (2017) had reported of other contexts. They took their point of departure in their particular circumstances and their knowledge of their students. What the teachers expressed then became a sort of justification of the organizational work of their teaching. During their joint participation in the sensemaking process, they addressed digital competence adequacy, that is, what Erstad et al. (2021) explained as relating to the pace of change in a digital society.

However, this paper shows an apparent discrepancy between transnational and national policies on digital competence and the results of this study. The difference was that policies, besides emphasizing competences in critical awareness and tool management, tended to underscore the importance of students being empowered (Redecker & Punie, 2017), fostered to participate in digital public democratic processes and spaces, that is, digital citizenship (European Commission, 2021), being able to manage net-based safety issues rather than avoiding them and having the opportunity to collaborate with others and solving various problems (European Commission et al., 2022). The Swedish national curriculum (National Agency for Education, 2022b) emphasizes digital competence, democracy and bildung as educational goals, even if it does not directly connect them to each other. Swedish government policy on digital competence states that “everybody should be familiar with digital tools and services, and have the ability to participate in digital development based on their prerequisites” (SKR 2017/18:47, p. 8). In this way, it refers to everybody’s participation in digital development, including students. Additionally, the Swedish national strategy on digitalization in education stresses the importance of actively participating in studies, a democratic society and, later, future work life (National Agency for Education, 2022a). The themes produced through the analysis in this study on critical awareness, tool management, creativity and avoidance partly suggest the need to take another stance in order for policy intentions to be met.

The results raise questions regarding this discrepancy between policies and what was identified in the sensemaking process. Before delving into this, I would like to stress that teachers are not to blame. Being a teacher in a digital society is challenging. According to Collin et al. (2018), teachers indicate difficulties when their assignment to provide core knowledge is contested by the transformation of social structures due to digitalization. Teachers can also experience difficulties connected to students’ access to digital content both in school and out of school (Löfving et al., 2023). The teacher participants only got glimpses of their students’ net-based out-of-school activities, which only painted part of the picture of their students’ experiences. The results should be read in this context and not in relation to whether teachers are doing right or wrong. They try their best to translate policy directives in the school organization, including its attendant hindrances or possibilities.

Thus, it is time to move on from a one-sided focus on individual teachers and pay attention to how schools as organizations can cater to their students to enable them to develop adequate digital competence, which is needed for core knowledge and various skills as citizens in a democratic digital society. Policymakers and school leaders are responsible for putting today’s questions on the agenda and acknowledging how to support PDC in relation to teachers’ competences in ”facilitating learners’ digital competence” (Redecker & Punie, 2017p. 16). This goes beyond the scope of the issues identified by Selwyn et al. (2020) regarding the logistics of digitalization and includes support for enhanced learning and the development of various skills in a digital society. If teachers’ assignments involve more than teaching core knowledge, which was questioned by some of the teachers in this study, then the requirements of this expanded task must be prioritized.

Given the issues raised in this study, further research is needed to examine what is meant by adequate digital competence and and who determines adequacy. Similarly, the results suggest that questions of how the boundary object of digital competence relates to particular policies in contexts should be pursued while also paying attention to the help that teachers need from school organizations to participate in sensemaking processes on students’ digital competence.

In conclusion, if students’ digital citizenship, which according to Choi (2016) targets democracy and empowerment, is to be considered part of students’ digital competence, standardized policies cannot cover all emerging issues in a changing society. The results of this study underline that students’ digital experiences gained from their net-based out-of-school activities need to be further considered, negotiated, and facilitated in school. This must be part of the new normal if students’ digital citizenship during childhood and adolescence is to be reckoned with.

## Data Availability

Data will be made available on reasonable request. The datasets generated during and analyzed during the current study are available from the corresponding author on reasonable request.
